# Evaluation of Genome-Wide Expression Profiles of Blood and Sputum Neutrophils in Cystic Fibrosis Patients Before and After Antibiotic Therapy

**DOI:** 10.1371/journal.pone.0104080

**Published:** 2014-08-01

**Authors:** Massimo Conese, Stefano Castellani, Silvia Lepore, Orazio Palumbo, Antonio Manca, Teresa Santostasi, Angela Maria Polizzi, Massimiliano Copetti, Sante Di Gioia, Valeria Casavola, Lorenzo Guerra, Anna Diana, Pasqualina Montemurro, Maria Addolorata Mariggiò, Crescenzio Gallo, Angela Bruna Maffione, Massimo Carella

**Affiliations:** 1 Department of Medical and Surgical Sciences, University of Foggia, Foggia, Italy; 2 Laboratory of Preclinical and Translational Research, IRCCS-CROB, Rionero in Vulture, Italy; 3 Medical Genetics Unit, IRCCS “Casa Sollievo della Sofferenza (CSS)” Hospital, San Giovanni Rotondo, Italy; 4 Centro di Riferimento Regionale Pugliese per la Fibrosi Cistica, Az. Univ. Osp. “Policlinico”, Bari, Italy; 5 Department of Biomedical Sciences and Human Oncology, Section of Pediatrics, University of Bari, Bari, Italy; 6 Biostatistic Unit, IRCCS “Casa Sollievo della Sofferenza (CSS)” Hospital, San Giovanni Rotondo, Italy; 7 Department of Biosciences, Biotechnologies and Biopharmaceutics, University of Bari, Bari, Italy; 8 Department of Biomedical Sciences and Human Oncology, Section of Hygiene, University of Bari, Bari, Italy; 9 Department of Clinical and Experimental Medicine, University of Foggia, Foggia, Italy; The Ohio State University, United States of America

## Abstract

In seeking more specific biomarkers of the cystic fibrosis (CF) lung inflammatory disease that would be sensitive to antibiotic therapy, we sought to evaluate the gene expression profiles of neutrophils in CF patients before treatment in comparison with non-CF healthy individuals and after antibiotic treatment. Genes involved in neutrophil-mediated inflammation, i.e. chemotaxis, respiratory burst, apoptosis, and granule exocytosis, were the targets of this study. Microarray analysis was carried out in blood and airway neutrophils from CF patients and in control subjects. A fold change (log) threshold of 1.4 and a cut-off of p<0.05 were utilized to identify significant genes. Community networks and principal component analysis were used to distinguish the groups of controls, pre- and post-therapy patients. Control subjects and CF patients before therapy were readily separated, whereas a clear distinction between patients before and after antibiotic therapy was not possible. Blood neutrophils before therapy presented 269 genes down-regulated and 56 up-regulated as compared with control subjects. Comparison between the same patients before and after therapy showed instead 44 genes down-regulated and 72 up-regulated. Three genes appeared to be sensitive to therapy and returned to “healthy” condition: phorbol-12-myristate-13-acetate-induced protein 1 (*PMAIP1*), hydrogen voltage-gated channel 1 (*HVCN1*), and β-arrestin 1 (*ARRB1*). The up-regulation of these genes after therapy were confirmed by real time PCR. In airway neutrophils, 1029 genes were differentially expressed post- vs pre-therapy. Of these, 30 genes were up-regulated and 75 down-regulated following antibiotic treatment. However, biological plausibility determined that only down-regulated genes belonged to the gene classes studied for blood neutrophils. Finally, it was observed that commonly expressed genes showed a greater variability in airway neutrophils than that found in blood neutrophils, both before and after therapy. These results indicate more specific targets for future interventions in CF patients involving respiratory burst, apoptosis, and granule exocytosis.

## Introduction

Cystic fibrosis (CF; OMIM 219700) is the most common lethal inherited disease in the Western world, with respiratory failure accounting for more than 80% of deaths from the disease, usually in the third or fourth decade of life. The hallmarks of CF lung disease are bacterial infections by opportunistic pathogens and chronic inflammation, progressing to obstructive lung disease and bronchiecstasis [1]. CF lung inflammatory disease is characterized by high concentrations of neutrophil chemokines, such as IL-8, and a sustained accumulation of polymorphonuclear neutrophils in the airways [2], [3].

Respiratory functional tests (RFTs) are the most established outcome measure for CF therapies and a key consideration in the advancement of treatments from phase 2 to phase 3 trials. Limitations of RFTs endpoints include the fact that they are relatively insensitive to early disease and have a very limited ability to detect regional heterogeneity of disease. Many of the measurements of surrogate endpoints, including RFTs, assess function rather than structure [4]. Simple and non-invasive biomarkers of this inflammatory process are urgently needed to monitor disease progression, identify exacerbations, and evaluate the efficacy of novel therapies [5]. Furthermore, there is a critical need for effective antimicrobial and anti-inflammatory therapies to mitigate disease in these individuals. Furthermore, the design of clinical trials in CF is hampered, in part, by the lack of sensitive measures of treatment response.

A systemic marker of lung inflammation has many advantages, because blood can be obtained from subjects of any age and disease severity, and may reflect the status of inflammation throughout the lung, rather than one segment, as is assessed by bronchoalveolar lavage, or heterogenous segments, as with sputum. Assessments in blood have included products of inflammation [6], neutrophil elastase 1-antiprotease complexes [7], C-reactive protein (CRP) [8]–[11], various cytokines and growth factors from serum or plasma [10], [12]–[15], and blood cells themselves [16]–[20]. The gene expression of peripheral mononuclear cells has been recently studied as a predictor of treatment response in CF [21] and in predicting reduced pulmonary infection [22], however the role of mononuclear cells (lymphocytes and monocytes) in the pathophysiology of CF lung disease has not been well established yet. Overall, systemic indicators of inflammation have low sensitivity and show only modest increases during acute exacerbations. Thus, to date, no systemic marker of treatment response, and in particular of neutrophilic inflammation, has been validated in CF for assessing the therapeutic outcome.

The main aims of this study are to understand if CF patients in acute exacerbation status display a transcriptome profile in their blood neutrophils different from that of control subjects' neutrophils, and to determine whether antibiotic treatment for an acute exacerbation can be described by a change in gene expression in blood neutrophils. A further aim of this study was to find out whether gene expression profiles differ in sputum neutrophils compared with blood neutrophils before and after antibiotic therapy. Previous studies have shown that airway-derived neutrophils are different from blood neutrophils, in terms of cytokine production [17], [23], functional and signaling pathways [24], [25], although not in gene expression profiles [26]. Thus, comparison of differential expression of neutrophil genes between blood and sputum samples may serve to generate additional hypotheses concerning disease pathogenesis, inflammatory response regulation, and new targets for CF therapy.

## Materials and Methods

### Patients

Sixteen subjects with CF (all F508del homozygous) were enrolled at the time of admission for a clinically diagnosed pulmonary exacerbation at a CF Regional Centre of Bari. The study was approved by the ethics committee of the Azienda Ospedaliera Universitaria “Policlinico” of Bari (n. 1373/CE/2012) and performed in accordance with the 1964 Declaration of Helsinki. Written informed consent from the adult study subjects or written consent from the next of kin, caretakers, or guardians on behalf of the enrolled children was obtained. Patients identified for enrollment met criteria for an exacerbation, defined as a deterioration in symptoms perceived by the patient and an increase in cough, sputum production, dyspnea, decline in forced expiratory volume in 1 sec (FEV_1_) compared with previous best, weight loss and fever [27]. All patients were treated with one or more antibiotics targeting their specific bacterial pathogens for 10 days on average. The study design utilizes within subject comparisons, such that each study subject served as their own control, following treatment with antibiotics. However, blood neutrophils were recovered also from seven healthy subjects matched for age and sex. Blood was drawn and sputum was collected at the initiation and the completion of intravenous antibiotic therapy. At each time-point, lung function tests (FEV_1_ and forced vital capacity [FVC]) were performed by spirometry and the following parameters were measured in plasma: CRP, whole blood counts (WBC) and percentage of polymorphonuclear neutrophils (PMNs).

### Neutrophil isolation

Blood was collected onto heparin (20 IU/ml) in BD Vacutainer tubes. Five ml of blood were mixed with five ml of Hemagel containing heparin. After sedimentation, the leukocytes were recovered and then layered on Lymphoprep (Sigma, St. Louis, MO, USA). The ratio was two volumes of leukocytes to one volume of Lymphoprep. After centrifugation for 25 min at 15°C and 500×g, the granulocyte layer were diluted in Hank's (M-Medical S.r.l., Milano, Italy) and then centrifuged once for 5 min at 300×g. Contaminating erythrocytes were lysed after a 5-min incubation of the resuspended cells at room temperature in 2 ml of sterile distilled water. Lysis was stopped by the addition of a large excess of Hank's, and the cells were washed and centrifuged for 10 min at 200×g. The purity of blood granulocytes, assessed by May-Grünwald-Giemsa staining of cytospins, was >95%. Viability was >95% as assessed by trypan blue staining. Complete blood counts were done to quantify cell numbers.

The sputum spontaneously coughed was collected in sterile cups from CF patients and was processed immediately. The sputum was added to 2.5 volumes of sterile saline solution and shaken vigorously. The washed sputum was then added to the same volume of Sputasol, a 0.1% dithiothreitol formulation (OXOID Ltd, Hampshire, UK), and the mixture was shaken vigorously at 37°C, and the incubation was continued until liquefaction of mucus was obtained. Samples were centrifuged for 3 min at 37×g to remove gross debris and the supernatants were centrifuged for 10 min at 253×g. Neutrophils in the pellet were then purified by positive selection after incubation with the anti-human CD66b-coated magnetic beads, in order to remove contaminating monocytes (Easy Sep Human Neutrophil Enrichment Kit, StemCell Technologies, Vancouver, Canada). Neutrophils were counted and their viability evaluated by the trypan blue dye exclusion test (>95%). The purity of the neutrophil suspension was assessed by May-Grünwald-Giemsa staining (>99%). In this part of the study, healthy subjects were not considered since the amount of sputum was not appropriate to isolate airway neutrophils. After each cell preparation, the RNA was immediately isolated by Trizol method, then purified using the RNeasy Mini Kit (Qiagen) according to manufacturer's protocol. Utilization of RNA samples obtained from blood and airway CF neutrophils is represented in Table S1.

### Microarray experiments and statistical analysis

All RNAs were quantified using the Thermo Scientific NanoDrop 1000 Spectrophotometer (NanoDrop technologies, Berlin, Germany) while the quality of each RNA was determined by running aliquots on the 2100 Bioanalyzer (Agilent Technologies, Waldbronn, Germany). Samples with a RNA integrity number <6.5 or a concentration <50 ng/µl were excluded from the study while samples with a RNA integrity number ≥6.5 and a concentration ≥50 ng/µl were labelled for GeneChip Human Gene 1.0 ST Array System (Affymetrix, Santa Clara, CA) that interrogates 28,869 well annotated genes by using an average of 26 probes per gene. 100 ng of total RNA were processed according to the GeneChip Whole Transcript (WT) Sense Target Labeling Assay as provided by the manufacturer. Briefly, a random priming method was used to generate cDNA from all RNA transcripts present in a sample. The random primers incorporate a T7 promoter sequence, which is subsequently used in an in vitro transcription to produce antisense cRNA fragments. Single stranded cDNA complementary to the cRNA is then produced, in the sense orientation, where a modified dUTP is incorporated instead of dTTP. The modified dUTP is subsequently recognised by the enzymes Uracil-DNA glycosylase (UDG) and human apurinic/apyrimidinic endonuclease 1 (APE 1) which will cut the DNA, resulting in fragmentation of the cDNA. Each DNA fragment is end-labelled with biotin using terminal deoxynucleotidyltransferase (TdT) before being hybridised to the arrays for 16 h at 45°C in a GeneChip hybridization Oven 640. Following hybridization and post hybridization washes the arrays were scanned using the AffymetrixGeneChip Scanner 3000 7G to generate the raw data (.CEL file). The QC steps of the experiment were performed using Expression Console (Affymetrix, Santa Clara, CA) software while the statistical analysis using Partek Genomic Suite (Partek Inc., St. Louis, MO). Briefly, raw intensity values were imported by setting up robust multiarray analysis (RMA) background correction, quartile normalization, and log transformation. In order to identify statistically significant genes, comparison of gene expression values for control vs pre-therapy was performed by unpaired t test and pre- vs post-antibiotic therapy by paired t-test; genes were identified as interesting if they displayed significant differential expression. A two-tailed p-values <0.05 was considered as statistically significant. After identifying the significant genes, each dataset (control vs pre-therapy and pre- vs post-therapy) was reduced to the only genes of interest in terms of biological plausibility, and checked against ANOVA. Furthermore, only genes above an absolute log expression ratio threshold of 1.4 were considered.

**Figure 1:**
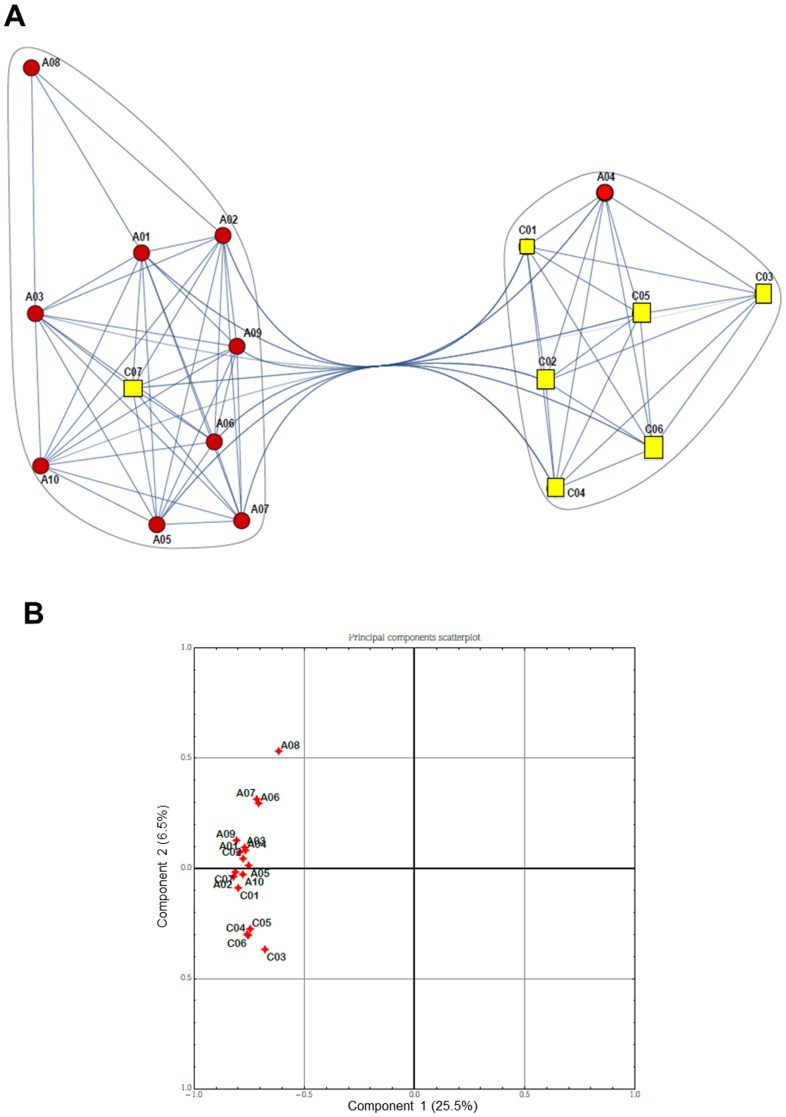
Comparison of global gene expression between controls and pre-therapy patients. A) Community graphs between controls (C, yellow squares) and pre-therapy (A, red circles) patients. Separation is not complete due to some information overlapping among samples. B) PCA scatterplot. Correlation between controls (C) and pre-therapy (A) patients with respect to the two first principal components computed with the Principal Component Analysis.

**Figure 2:**
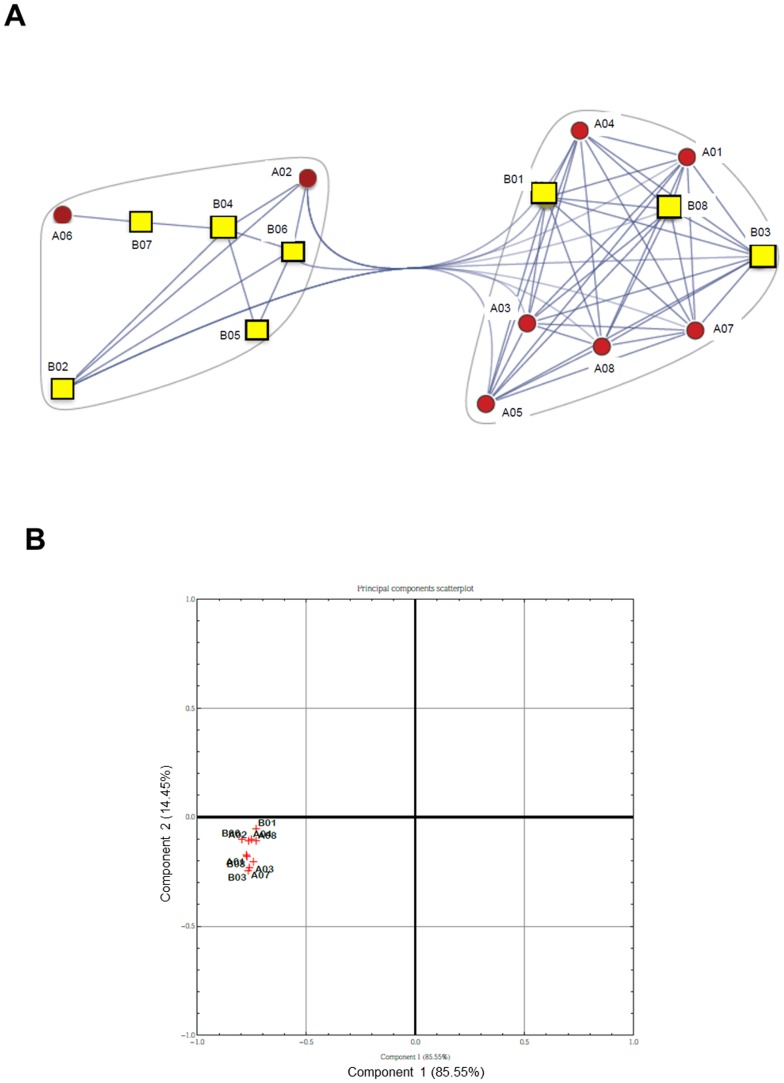
Comparison of global gene expression between patients pre- and post-therapy. A) Community graphs between pre- (A, red circles) and post- (B, yellow squares) therapy patients. Separation is not complete due to some information overlapping among samples. B) PCA scatterplot. Correlation between pre- (A) and post- (B) therapy patients with respect to the two first principal components computed with the Principal Component Analysis.

Then clustering was performed on genomic samples in order to identify subtypes among the patients by means of a “correlation network”, which was built from the reduced datasets connecting those patients displaying correlated expression (above a given threshold). In this network, the numerical weights on the edges are the absolute correlation coefficients, while the nodes represent the analyzed samples. We named the obtained correlation networks “communities”, with many edges joining vertices of the same community and comparatively few edges joining vertices of different communities [28]. The control vs pre-therapy and pre- vs post-therapy datasets were further investigated through Principal Components Analysis (PCA) [29] as it is an excellent method for expression data and allowed us to summarize the ways in which gene expression profiles over samples vary under different conditions. We also examined the communities obtained by observing correlations between samples, and show how they are manifested in principal component space (see Figures 1B and 2B) reducing multi-dimensional data and determining the key variables in a multidimensional data set that explain the differences in the observations.

In order to analyze differences between gene expression between airway and blood neutrophils, log-transformed data obtained from each complete dataset, pre-therapy (n = 10, blood neutrophils; n = 4, airway neutrophils) and post-therapy (n = 8, blood neutrophils; n = 4, airway neutrophils) were scrutinized for common genes. The expression values were averaged and compared with the mean values of the corresponding blood control neutrophil dataset (n = 7) for identifying up- and down-regulated genes.

### Real time PCR validation

Microarray expression data were confirmed by real-time polymerase chain reaction (RT-PCR), utilizing all sufficient remaining RNA for analysis. First strand cDNA was made from 1 µg total RNA using a QuantiTect Reverse Transcription Kit (Qiagen, Valencia, CA). Quantitative real-time PCR was performed using a total reaction volume of 20 µl, containing 7.9 µl of diluted cDNA in H_2_O, 1.1 µl of the specific Taqman gene expression assay (Applied Biosystems, Foster City, CA) and 11 µl of the 2X Taqman Universal Master Mix (Applied Biosystems). ID assay for *HVCN1*: Hs00260697_m1; for *PMAIP1*: Hs00560402_m1; for *ARBB1*: Hs00244527_m1;for *DOM3Z*: Hs00364127_g1. PCR was carried out in a StepOne Plus Real Time PCR System (Applied Biosystems), using 40 cycles of 95°C for 15 seconds, followed by 60°C for 1 minute, with a 10 minute 95°C initial soak. Each measurement was made in triplicate and expressed relative to the detection of the housekeeping gene, hypoxanthine guanine phosphoribosyltransferase (HPRT, ID assay: Hs99999909_m1). For quantitative RT-PCR, statistics were performed in StatView (SAS, Cary, NC), utilizing paired t-tests, and significance at P<0.05.

## Results

### Patient characteristics

Sixteen CF patients were enrolled at the moment of hospitalization for an acute exacerbation (Table 1). On the same day, one or more patients were studied, together with overall 7 healthy subjects, matched for age and sex. Antibiotic treatment lasted 10 days in average. The mean age of the CF patients recruited in this study was 25.8 years, range 11–42. Males were 12 (75%). Sputum culture isolated *Pseudomonas aeruginosa* from nine patients, *Staphylococcus aureus* from six patient, *Burkholderia cenocepacia* from two patients, *Aspergillum fumigatus* from one patient, *Hemophilus influenzae* from one patient, *Scedosporium apiospermum* from one patient. Antibiotic choice varied between patients, depending on microbiological susceptibility testing and clinical response. Initial antibiotic choices (one, two to three antibiotics per patient) included ciprofloxacin (n = 7), tobramycin (n = 5), azithromycin (n = 5), amikacin (n = 2), ceftazidim (n = 2), meropenem (n = 2), minociclin (n = 2), teicoplanin (n = 1), tigecyclin (n = 1), sulfametoxazole/trimetoprim (n = 1). Patients with fungal infections were treated also with itroconazole (for *A. fumigatus*) and with voriconazole (for *S. apiospermum*).

**Table 1:** Demographics, sputum microbiology and antibiotic therapy of CF patients.

Patient #	Sex	Age (years)	Sputum microbiology	Antibiotic therapy	FEV_1_ (% predicted)		FVC (% predicted)		WBC (*1000/mm^3^)		PMN (% of WBC)		CRP (mg/dl)	
					Pre- therapy	Post-therapy	Pre- therapy	Post-therapy	Pre- therapy	Post-therapy	Pre- therapy	Post-therapy	Pre- therapy	Post-therapy
1	M	36	*S. aureus*	Ceftazidim Tobramycin	28.9	37.7	43.3	54.6	10.69	9.41	66	54.7	3.6	1.4
2	M	27	*P. aeruginosa* (mucoid) *A. fumigatus*	Ceftazidim Tobramycin	25.7	31.5	37.0	44.9	10.37	6.54	74.8	56.9	9.7	3.2
3	M	30	*P. aeruginosa S. apiospermum*	Sulfametoxazole/Trimetoprim Azithromycin Tobramycin	17.5	17	37.2	35.6	15.63	15.05	72.7	76.4	23.1	32
4	M	30	N/A	Azitrhomycin Tobramycin	35.8	41.6	52.2	62.5	6.90	8.08	55.5	54.2	3.4	2.8
5	F	11	N/A	Ciprofloxacin Azithromycin	105.7	99.3	114.6	107.7	6.43	4.63	39.9	37.6	1.4	1.9
6	M	16	*P. aeruginosa, S. aureus*	Ciprofloxacin	78.0	72.4	80.4	77.0	8.66	8.71	57.7	59.1	2,2	1.4
7	M	29	*P. aeruginosa*, *S. aureus*, *H. influenzae*	Meropemen Amikacyin	19.1	17.7	31.4	25.6	9.68	6.79	81.6	62.5	27.6	N/A
8	M	17	*P. aeruginosa*	Ciprofloxacin Azithromycin	65.4	95.9	84.7	104.1	10.20	8.87	60.1	58.1	24.0	3.2
9	F	29	*B. cepacia*	Minociclin	20.7	22.6	29.9	32.4	11.87	12.17	60.7	62.3	103.8	73.1
10	M	42	*P. aeruginosa*	Ciprofloxacin Azithromycin	17.8	19.8	33.9	31.1	8.21	6.94	60.5	62.8	N/A	N/A
11	M	24	N/A	N/A	50.4	67.9	64.9	78.4	4.37	8.33	55.6	70.7	12.1	2.9
12	M	19	*P. aeruginosa, S. aureus*	Ciprofloxacin	57.0	59.7	79.0	84.0	10.33	9.85	57.1	42.9	14.6	6.3
13	F	16	*S. aureus*	Minociclin Teicoplanin	32.1	34	40.8	48.3	7.98	9.56	45.0	40.5	13.8	2.5
14	F	39	*P. aeruginosa*	Amikacin Meropenem Ciprofloxacin Tigecycline	40.1	46.6	52.6	58.9	7.6	7.7	61.7	60.3	5.0	2.0
15	M	28	*S. aureus B. cepacia*	Ceftazidim Tobramycin	62.8	63.8	N/A	86.5	7.05	12.3	57.1	57.7	3.8	1.4
16	M	21	*P. aeruginosa*	Ciprofloxacin	92.5	100.0	107.0	109.0	10.29	8.23	64.5	47.4	4.2	N/A

N/A: not available.

### Blood inflammatory parameters and respiratory function tests

Hematologic parameters and RFTs (FEV_1_ and FVC) at the start were compared with those at the end of a course of antibiotics. Following completion of treatment, FEV_1_ and CRP increased and decreased significantly after treatment, respectively, and FVC showed a borderline significant increase, while there were no significant differences in WBC and % PMNs as compared with pre-treatment values (Table 2).

**Table 2:** Respiratory function tests and hematologic parameters before and after antibiotic therapy of all patients.

	Pre-therapy	Post-therapy	P value
WBC (*1000/mm^3^)	8.4 (7.2, 10.4)	8.8 (7.1, 9.8)	0.8564
PMN (% of WBC)	60.3 (56.0, 65.6)	57.9 (49.1, 62.4)	0.1272
CRP (mg/dl)	9.7 (3.5, 18.8)	2.8 (1.6, 4.7)	0.0105
FEV_1_ (% of predicted)	37.9 (21.9, 64.7)	44.1 (24.8, 71.3)	0.0214
FVC (% of predicted)	52.2 (37.0, 80.4)	58.9(35.6, 84.0)	0.0554

Values are shown as median (25th percentile, 75th percentile). P values were calculated using the Wilcoxon signed rank test.

### Gene expression profile of blood PMNs

A net separation between control subjects and CF patients before therapy, except for one subject in each group, was evident by the gene clustering presentation of the microarray, which was confirmed by the principal component analysis (Figure 1). From the core gene set, 581 genes were differentially expressed in CF patients before therapy relative to control subjects at a fold change threshold of 1.4 (Table S2). Out of these genes, 269 genes were down-regulated and 56 up-regulated as compared with control (considering as cut-off p<0.05). Genes with biological plausibility were categorized according to gene ontology and biological process. We focused our attention on genes whose products are involved in apoptosis, cell adhesion, chemotaxis, inflammation, and immune response (Table 3 and Table 4). The genes involved in the apoptotic response and down-regulated were pro-apoptotic, suggesting that blood neutrophils in CF patients may present a reduced apoptosis compared with non-CF subjects. The genes comprised in the inflammatory and immune response were down-regulated as well. Also the genes involved in cell adhesion, intercellular interaction, cytoskeletal arrangement, and respiratory burst were mostly down-regulated.

**Table 3:** Up-regulated genes in blood PMN of CF patients vs. controls subjects with biological plausibility.

Biological process	Accession number	Gene coding for	p-value*	Fold change
Apoptosis	NM_000212	ITGB3 (integrin, beta 3)	0.044	2.09
Immune response	NM_000610	CD44	0.0009	1.95
Inflammation, apoptosis	NM_004895	NLRP3 (NLR family, pyrin domain containing 3)	0.011	2.33
Inflammation, signal transduction	NM_020370	GPR84 (G protein-coupled receptor 84)	0.007	2.37
Signal transduction	NM_002184	IL6ST (interleukin 6 signal transducer; gp130;oncostatin M receptor)	0.034	1.74

*from unpaired t-test.

**Table 4:** Down-regulated genes in blood PMN of CF patients vs. control subjects with biological plausibility.

Biological process	Accession	Gene coding for	p-value*	Fold change
Adhesion	NM_001627	ALCAM (activated leukocyte cell adhesion molecule)	<0.0001	−1.83
Apoptosis	NM_181861	APAF1 (apoptotic peptidase activating factor 1)	0.021	− 2.27
Apoptosis	NM_033004	NLRP1 (NLR family, pyrin domain containing 1)	0.001	−1.52
Apoptosis	NM_021127	PMAIP1 (phorbol-12-myristate-13-acetate-induced protein 1)	0.016	−1.66
Apoptosis	NM_004938	DAPK1 (death-associated protein kinase 1)	0.007	−1.80
Cell adhesion, autophagy	NM_014385	SIGLEC7 (sialic acid binding Ig-like lectin 7)	0.001	−1.63
Cytoskeleton complex	NM_015326	SRGAP2 (SLIT-ROBO Rho GTPase activating protein 2)	0.008	− 1,57
Immune response, apoptosis	NM_001561	TNFRSF9 (tumor necrosis factor receptor superfamily, member 9)	0.0008	− 2.14
Immune response	NM_001040107	HVCN1 (hydrogen voltage-gated channel 1)	0.001	−1.87
Immune response	NM_014238	KSR1 (kinase suppressor of ras 1)	0.009	−1.66
Immune response	NM_000442	PECAM1 (platelet/endothelial cell adhesion molecule)	0.013	−1.53
Immune response	NM_006863	LILRA1 (leukocyte immunoglobulin-like receptor, subfamily A)	0.022	−1.71
Immune response	NM_000607	ORM1 (orosomucoid 1)	0.045	−2.62
Immune response, signaling	NM_007261	CD300a	0.025	−1.67
Inflammation	NM_020530	OSM (oncostatin M)	0.003	−1.69
Inflammation	NM_001837	CCR3 (chemokine (C-C motif) receptor 3)	0.009	−2.92
Inflammation	NM_005211	CSF1R (colony stimulating factor 1 receptor)	0.016	−1.75
Inflammation	NM_006639	CYSLTR1(cysteinyl leukotriene receptor 1)	0.009	−1.66
Oxidative burst	NM_001693	ATP6V1B2 (ATPase, H+ transporting, lysosomal)	0.037	−1.51
Pathogen defence	NM_000081	LYST (lysosomal trafficking regulator)	0.026	− 1.86
Signal Transduction	NM_006990	WASF2 (WAS protein family, member 2)	0.009	− 1.41
Signal transduction	NM_004041	ARRB1 (Arrestin beta1)	0.004	− 1.79
Signal transduction	NM_207345	CLEC9A (C-type lectin domain family 9)	0.008	− 1.51
Signal transduction	NM_006779	CDC42EP2 (CDC42 effector protein (Rho GTPase binding 2)	0.016	−1.68

*from unpaired t-test.

Due to scarcity of mRNA recovered from blood neutrophils of 2 patients in post-treatment, the comparison between pre- and post-therapy was possible for 8 patients. Gene clustering and principal component analysis revealed that a clear distinction between patients before and after antibiotic therapy was not possible (Figure 2). From the core gene set, 1084 genes were differentially expressed in CF patients after therapy relative to the same patient before therapy at a fold change threshold of 1.4 (Table S3). Out of these genes, 72 genes were up-regulated and 44 down-regulated following antibiotic treatment (with a cut-off of p<0.05). As in the other comparison, we focused on genes with biological plausibility, finding that only up-regulated genes were comprised in this selection (Table 5). In contrast to the pattern outlined above in the same patients before therapy, antibiotic treatment determined up-regulation of genes involved in the apoptosis, cell adhesion, chemotaxis, inflammation and immune response. It is worth note that some inflammatory and immune (*IFI6*, *GBP1*, *GBP4*, *OAS1*, *OAS2*, and *OAS3*) genes were regulated by interferons. Neutrophils have been described as non-professional antigen presenting cells, expressing MHC and co-stimulatory molecules in certain conditions, and likely functioning as immunosuppressive cells [30]. Whether this can be seen as a positive effect on the adaptive immune response in these patients is not known at the moment.

**Table 5:** Up-regulated genes in blood PMN of CF patients post-therapy vs. pre-therapy with biological plausibility.

Biological process	Accession	Gene coding for	p-value*	Fold change
Apoptosis	NM_001143676	SGK1 (serum glucocorticoid regulated kinase 1)	0.0013	1.49
Apoptosis	NM_021127	PMAIP1 (Noxa)	0.0036	2.00
Apoptosis	NM_002038	IFI6 (interferon, alpha-inducible protein 6)	0.0272	1.58
Apoptosis	NM_017523	XAF1 (XIAP associated factor 1)	0.0210	1.82
Cytoskeleton complex	NM_015326	SRGAP2 (SLIT-ROBO Rho GTPase activating protein 2)	0.0005	1.77
Immune response	NM_001040107	HVCN1 (hydrogen voltage-gated channel 1)	<0.0001	1.49
Immune response	NM_009587	LGALS9 (lectin, galactoside-binding, soluble, 9)	0.0106	1.48
Immune response	NM_002535	OAS2 2′-5′-(oligoadenylate synthetase 2)	0.0272	1.94
Immune response	NM_016816	OAS1 2′,5′-(oligoadenylate synthetase 1)	0.0331	1.76
Innate immune response	NM_006187	OAS3 2′-5′-(oligoadenylate synthetase 3)	0.0022	1.69
Lysosome system	NM_002355	M6PR (mannose-6-phosphate receptor)	0.0304	1.60
Signal transduction	NM_000024	ADRB2 (adrenoceptor beta 2, surface)	0.0011	1.42
Signal transduction	NM_004041	ARRB1 (Arrestin beta1)	0.0036	1.49
Signal transduction	NM_002053	GBP1(guanylate binding protein 1)	0.0084	1.42
Signal transduction	NM_052941	GBP4 (guanylate binding protein 4)	0.0017	1.72
Signal transduction	NM_000885	ITGA4 integrin, alpha 4 (antigen CD49D, alpha 4 subunit of VLA-4)	0.0043	1.72
Signal transduction	NM_001781	CD69	0.0497	2.11
Signal transduction	NM_018284	GBP3 (guanylate binding protein 3)	0.0033	1.58
Signal transduction	NM_003874	CD84	0.0482	1.59
Transcription	NM_004430	EGR3 (early growth response 3)	0.0034	1.55
Transcription	NM_001964	EGR1 (early growth response 1)	0.0334	2.23

*from paired t-test.

### Confirmation of microarray data by real time PCR

**Figure 3:**
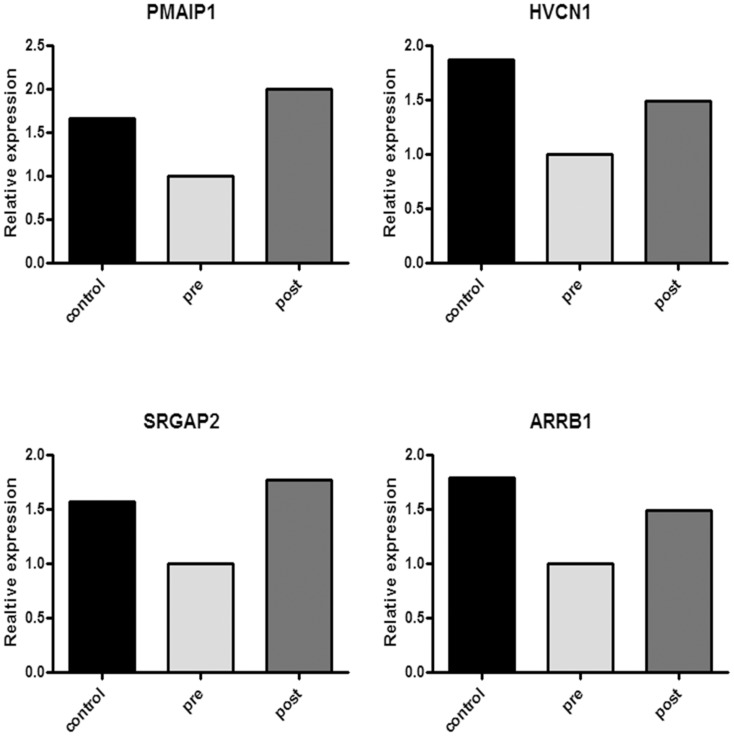
Comparison of gene expression obtained by microarray data for *HVCN1*, *PMAIP1, SGRAP2*, and *ARRB1* in healthy controls and CF patients before and after therapy. These four genes were downregulated in CF patients in comparison with controls and returned towards “healthy” condition after therapy. Histograms were generated setting gene expression in CF patients before therapy at 1.

**Figure 4:**
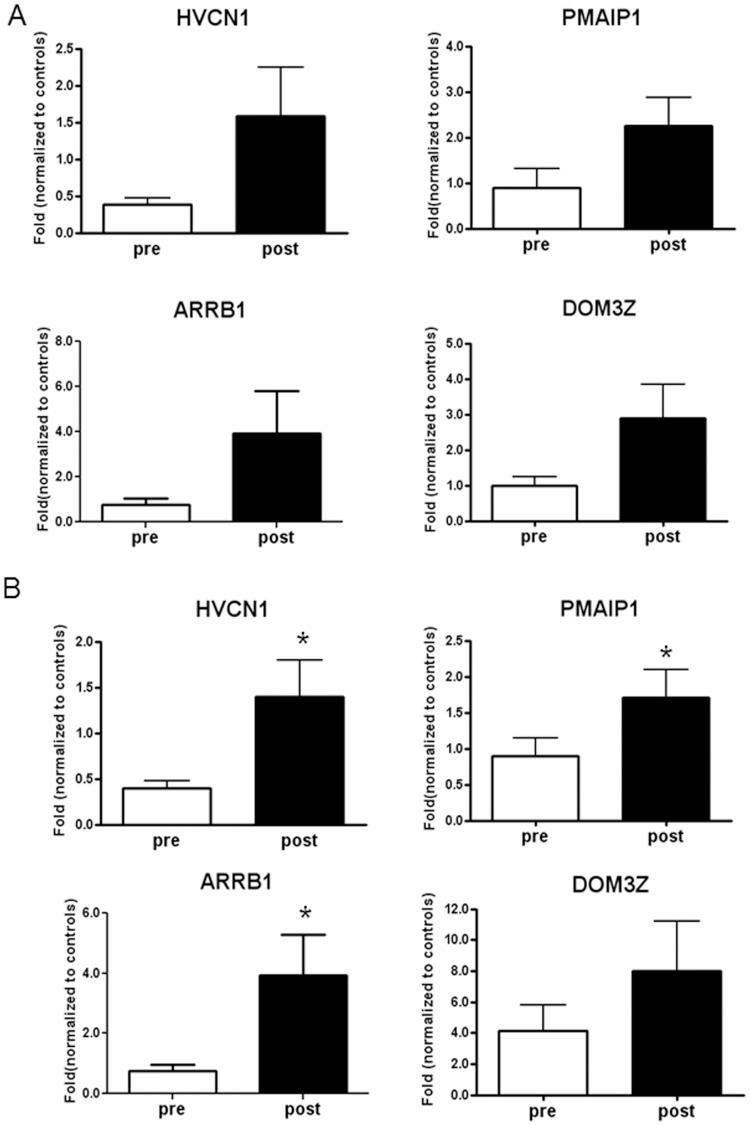
Real-time polymerase chain reaction (RT-PCR) on *HVCN1*, *PMAIP1*, *ARRB1* and *DOM3Z* in blood neutrophils obtained from CF patients pre- and post-therapy confirming microarray expression data. A) Data were generated by comparing 5 healthy controls and 5 CF patients before and after therapy setting gene expression in healthy controls at 1. *HVCN1* was less expressed in CF patients as compared to controls and its expression increased after therapy to values above those of controls. *ARRB1* and *PMAIP1* in CF patients before therapy showed similar levels of the control subjects but nevertheless increased after therapy above values of controls. B) Validation experiment carried out in 10 healthy subjects and 10 CF patients before and after antibiotic treatment. Each measurement was made in triplicate and expressed relative to the detection of the housekeeping gene *HPRT*. For quantitative RT-PCR, statistics were performed by utilizing paired t-test. *P<0.05, post vs. pre.

Interestingly, four genes appeared to be sensitive to therapy and returned towards “healthy” conditions: phorbol-12-myristate-13-acetate-induced protein 1 (*PMAIP1*, also named *NOXA*), hydrogen voltage channel 1 (*HVCN1*), slit-ROBO RhoGTPase activating protein 2 (*SRGAP2*), and β-arrestin 1 (*ARRB1*) (Figure 3). Since we did not find a link between the *SRGAP2* gene and human neutrophils' expression and function in literature, we validated microarray data by real time PCR on *PMAIP1*, *ARRB1*, and *HVCN1* on five CF subjects. By comparison, we analysed also *DOM3Z* that was down-regulated in CF patients as compared to controls (p = 0.001; fold change of 1.72) and was up-regulated after treatment (p = 0.0098; fold change of 1.13). Raw data were normalised to the control subjects, put equal to 1 and expressed as fold-change. *HVCN1* was less expressed in CF patients as compared to controls and its expression increased after therapy to values above those of controls (Figure 4A). *ARRB1* and *PMAIP1* in CF patients before therapy showed similar levels of the control subjects but nevertheless increased after therapy above values of controls. *DOM3Z* behaved in similar fashion of *PMAIP1*, *ARRB1* and *HVCN1*. In order to further validate real time PCR data, we performed this analysis on other five control subjects and four CF patients evaluated at the time of acute exacerbation and after treatment (Table S1). Figure 4B shows cumulative data confirming data and gaining statistical significance.

### Gene expression profile of airway neutrophils

**Figure 5:**
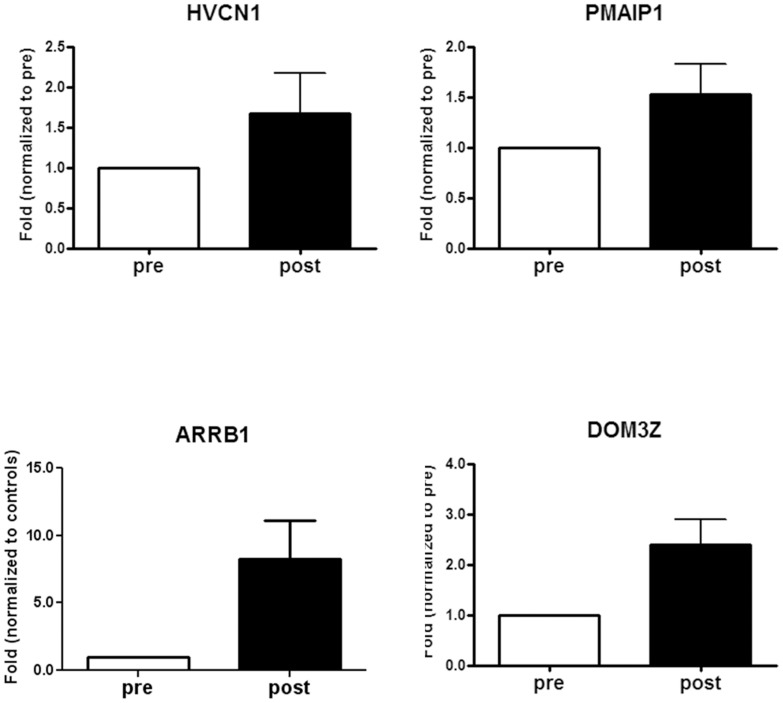
Real time PCR data on *HVCN1*, *PMAIP1*, *ARRB1* and *DOM3Z* in sputum neutrophils obtained from 6 CF patients before and after therapy. Gene expression in CF neutrophils before therapy was set at 1. Each measurement was made in triplicate and expressed relative to the detection of the housekeeping gene *HPRT*. For quantitative RT-PCR, statistics were performed utilizing paired t-test. All four genes were up-regulated in airway PMN after therapy but did not reach statistically significance.

Comparison of airway neutrophils obtained from a subset of CF patients before and after therapy (n = 4) showed that, at a fold change threshold of 1.4, 1029 genes were differentially expressed (Table S4). Of these, 30 genes were up-regulated and 75 down-regulated following antibiotic treatment (with a cut-off of p<0.05). However, biological plausibility determined that only down-regulated genes belonged to the gene classes studied for blood neutrophils (Table 6). In this list, the four genes highlighted for blood PMN are not present; nevertheless, as internal control for real time PCR data, we analysed *HVCN1*, *PMAIP1*, *ARRB1* and *DOM3Z* expression in airway neutrophils from 6 patients (Table S1). Figure 5 shows that all four genes were up-regulated in airway PMN after therapy but did not reach statistical significance.

**Table 6:** Down-regulated genes in airway PMN of CF patients post-therapy vs. pre-therapy with biological plausibility.

Biological process	Accession	Gene coding for	p-value*	Fold change
Apoptosis	NM_014143	CD274	0.0043	−2.22
Apoptosis	NM_033306	CASP4 (caspase 4)	0.0176	−1.68
Apoptosis	NM_004536	NAIP (NLR family, apoptosis inhibitory protein)	0.0323	−2.08
Immune response	NM_000544	TAP2 (transporter 2, ATP-binding cassette, sub-family B)	0.0012	−1.42
Immune response	NM_014879 14	P2RY14 (purinergic receptor P2Y, G-protein coupled)	0.0113	−1.56
Immune response	NM_006573	TNFSF13B (tumor necrosis factor (ligand) superfamily, member 13b)	0.0116	−1.56
Immune response	NM_003004	SECTM1 (secreted and transmembrane 1)	0.0240	−1.53
Immune response	NM_002198	IRF1 (interferon regulatory factor 1)	0.0345	−1.84
Inflammation	NM_024807	TREML2 (triggering receptor expressed on myeloid cells-like 2)	0.0003	−1.40
Inflammation	NM_006639	CYSLTR1 (cysteinyl leukotriene receptor 1)	0.0070	−1.76
Inflammation	NR_024168	TLR4 (toll-like receptor 4)	0.0178	−1.43
Inflammation	NM_020370	GPR84 (G protein-coupled receptor 84)	0.0247	−1.81
Inflammation	NM_000062	SERPING1 (serpin peptidase inhibitor)	0.0459	−1.51
Signal transduction	NM_016511	CLEC1a (C-type lectin domain family 1)	0.0010	−1.45
Signal transduction	NM_002053	GBP1 guanylate binding protein 1, interferon-inducible,	0.0021	−2.42
Signal transduction	NM_207345	CLEC9A (C-type lectin domain family 9)	0.0052	−1.43
Signal transduction	NM_005419	STAT2 (signal transducer and activator of transcription 2)	0.0079	−1.90
Signal transduction	NM_007315	STAT1 (signal transducer and activator of transcription 1)	0.0083	−1.81
Signal transduction	NM_003608	GPR65 (G protein-coupled receptor 65)	0.0117	−1.62
Signal transduction	NM_018284	GBP3 (guanylate binding protein 3)	0.0186	−1.81
Signal transduction	NM_004120	GBP2 (guanylate binding protein 2, interferon-inducible)	0.0216	−1.51
Signal transduction	NM_052941	GBP4 (guanylate binding protein 4)	0.0313	−1.67

*from paired t-test.

### Comparison between blood and airway neutrophils

**Figure 6:**
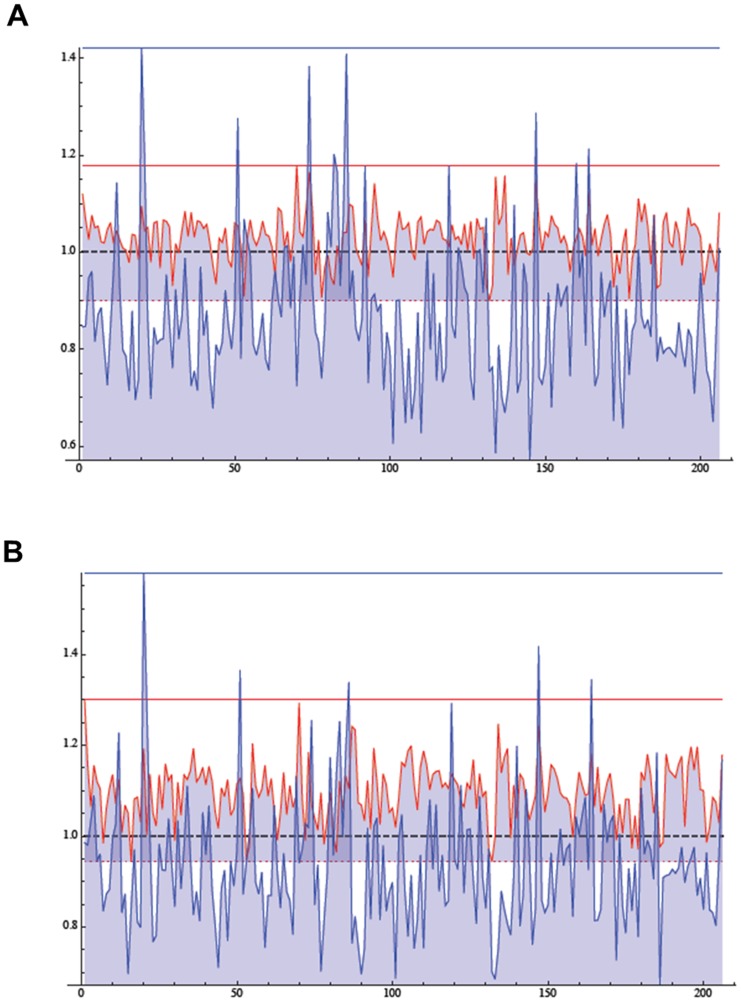
Commonly expressed genes in blood and airway neutrophils in comparison with control blood neutrophils. A) List-plot graph representing the average expression of 206 genes in blood (red line) and airway (blue line) neutrophils in patients pre-therapy. The average expression of control neutrophils is put equal to 1. Blood neutrophils ratios range from 0.899 to 1.179, while airway neutrophils ratios range from 0.571 to 1.422. B) List-plot graph representing the average expression of 206 genes in blood (red line) and airway (blue line) neutrophils in patients post-therapy. The average expression of control neutrophils is put equal to 1. Blood neutrophils ratios range from 0.944 to 1.299, while airway neutrophils ratios range from 0.673 to 1.579.

There are 206 genes common to the blood and airway datasets (Table S5). The average expression in the samples obtained from pre-therapy patients was compared for each gene with that of control blood neutrophils, not having sputum neutrophils from healthy individuals. From this comparison, 30 genes and 176 genes were up-regulated and down-regulated in airway neutrophils as compared to control neutrophils, respectively. On the other hand, 156 genes were up-regulated and 50 were down-regulated in blood neutrophils in comparison with control neutrophils. The list-plot graph (obtained by plotting and joining each common gene's fold-change value) shows this difference in the gene expression relative to the 206 genes, displaying a greater variability among airway genes (Figure 6A). In airway neutrophils obtained from CF patients post-therapy, 154 genes and 52 genes were found down-regulated and up-regulated, respectively. In airway neutrophils post-therapy, we found that 186 and 20 were up-regulated and down-regulated, respectively. Again, this difference is illustrated by the list-plot graph, evidencing again a greater variability in airway neutrophils (Figure 6B).

## Discussion

In CF, pulmonary exacerbations are defined based on increased symptoms, decrements in lung functions, and radiographic changes and are intrinsically a subjective judgment of physicians [31]. Moreover, one-quarter of patients fail to recover their previous baseline lung function despite therapy, likely because current management is suboptimal [32]. Biomarkers reflective of disease activity in pulmonary exacerbations have the potential to improve patient care. Systemic biomarkers monitoring inflammation are ideal because they could reflect the status of disease activity and severity throughout the entire lung, as opposed to one region as in the case of sputum. Although blood-based biomarkers have been widely studied in CF pulmonary exacerbations, only CRP consistently correlated with disease activity, with increases from stable to exacerbation state and decreases in response to therapy [11]. However, no systemic biomarker, including CRP, has been validated in a clinical trial supporting its clinical usefulness beyond routine clinical assessment. Thus, further research to find out more robust and sensitive biomarkers reflecting the lung disease activity and severity is needed.

In this study, we began to approach the systemic biomarker field by a genome-wide evaluation of gene expression in blood neutrophils, the principal immune cell involved in the CF lung inflammation. Our approach was to reduce the dimensions of data in order to focus on a very small but significant number of genes with a limited set of patient samples. This was done by employing differential expression analysis and subsequently verified by PCA. Data presented in this paper show that in blood neutrophils obtained from CF patients (all homozygous for the mutation F508del) there is an overall genic down-regulation compared with healthy subjects, including genes encoding for proteins involved in apoptosis, cell adhesion, inflammatory and immune response (Table 3). Antibiotic therapy treatment for 10 days reversed this figure to genic up-regulation (Table 4). These results suggest that blood neutrophils have a defect in apoptosis and activation, a condition brought to “healthy” status by antibiotic therapy.

Previously, Adib-Conquy and colleagues [26] published microarray analysis of 1050 genes in blood neutrophils collected from CF patients devoid of bacterial colonization and compared them with healthy subjects. Their list of upregulated and downregulated genes does not include genes found in the present study. This discrepancy might be ascribed to various differences between the two studies: just one of their five patients was F508del/F508del, the mean age was of 10.7 years, the mean FEV_1_ was 55% of predicted, all parameters which differ substantially from ours. Finally, our patients were all colonized by *P. aeruginosa* and other bacterial species, causing a comorbidity absent in the previous report. Thus, our study is the first to show genome-wide gene expression profiles of neutrophils of CF patients colonized by bacteria in their airways and in a clinical condition of acute exacerbation.

Recent work has focused on CF blood mononuclear cells in order to identify circulating transcripts as biomarkers of the treatment for an acute exacerbation. Saavedra et al. [21] found that 10 genes significantly changed with therapy and that three genes (CD64, ADAM9, and CD36) enhanced the predictive discriminating value of FEV_1_ alone. However, seven of the 10 genes are not specific to CF and they need further evaluation as their role and biomarker status in the CF disease. Interestingly, the same group has recently validated the 10 genes in a study on the whole blood in a cohort of CF patients treated for acute exacerbations [22]. Their findings show that six out of 10 genes strongly predicted a reduction in airway bacterial load beyond FEV_1_ and CRP, adding specificity in predicting reduced pulmonary infection. The six genes were not coincident with our three-genes panel sensitive to treatment for an acute exacerbation. This may reflect that these six genes are related more to mononuclear cells than to neutrophils.

One of the aims of this work is to find a set of genes which is differentially transcribed in CF as compared to “healthy” condition. This “CF signature” could be useful to identify CF patients unequivocally and start with therapy as soon as possible. A net separation between CF patients and healthy controls was obtained (Figure 1). A caveat to these findings is that, although we define a set of regulated genes in CF patients at the onset of an acute exacerbation, this clinical condition is not representative of the initial steps of disease, thereby further studies with different cohorts of patients in various clinical conditions are needed. It is not surprising that a distinct separation could be not possible when comparing patients before and after treatment (Figure 2), suggesting that patients can respond differently to antibiotic treatment for an acute exacerbation.

The sensitivity to therapy of the four genes (*HVCN1*, *PMAIP1*, *SRGAP2*, and *ARRB1*) is worthy of further investigation in the clinical setting. A higher number of patients is needed for studies aimed to prediction of acute exacerbation and follow up. Of the four genes, one (*SRGAP2*) was not further examined by real time PCR because of lack of data on its expression and function in neutrophils. Nevertheless, it deserves some attention, since *SRGAP2* is a Rho GTPase activating protein involved in actin dynamics [33] and has been shown to be needed for cell migration [34]. As for the other three genes, they are involved in apoptosis, respiratory burst and granule exocytosis, all biological process which are involved in neutrophil-mediated response. Here follows a brief discussion of what is known about their protein product and function in CF.

Noxa is a member of Bcl-2 family of proteins, a critical mediator of the p53-dependent apoptosis [35] and has been implicated in hypoxia-induced apoptosis [36]. Noxa is also likely involved in apoptosis of virus-infected cells to limit viral dissemination [37]. Recently, a strong pro-apoptotic role of Noxa in the final steps of neutrophil differentiation from progenitors has been described [38]. A delayed apoptotic response has been described in blood neutrophils isolated from CF patients [39]–[41]. Our data are in line with a reduced apoptosis of CF circulating neutrophils and its recovery after an antibiotic treatment. A reduced apoptotic response in acute exacerbation might be correlated with longer neutrophil survival and more damage to the airways.

During phagocytosis, *HVCN1* is involved in maintaining NADPH oxidase activity by preventing acidification to an intracytosolic pH low enough to inhibit NADPH oxidase [42]. A low level of *HVCN1* transcripts in CF patients before therapy as compared to non-CF subjects is suggestive of an impaired oxidative burst and pathogen survival in this condition. However, the respiratory burst in CF neutrophils has been demonstrated to be extremely variable as compared to “healthy” neutrophils [39], [43], [44]. To the best of our knowledge, HVCN1 protein expression and function have not been studied in CF neutrophils and our data therefore open a novel avenue in the study of neutrophil antibacterial function in CF lung disease.

*ARRB1* is a scaffolding protein involved in platelet-activating factor-induced endocytosis and cytoskeleton rearrangement [45]. β-arrestins may also be required for activating signaling pathways leading to exocytosis of primary and secondary granules in neutrophils [46]. Like HVCN1,the ARRB1 protein has not been investigated in its expression and function in CF neutrophils.

Functional studies are needed to elucidate which effect *ARRB1*, *HVCN1* and *PMAIP1* mRNA fluctuations exert on the granule exocytosis, respiratory burst, as well on the apoptotic response. The sensitivity of *ARRB1*, *PMAIP1* and *HVCN1* to the antibiotic treatment makes these three genes promising candidates for the evaluation of the response to therapy, although this should be substantiated by studies correlating these transcript to respiratory functional tests or follow up.

Sputum neutrophils were found to have a limited set of expressed genes in common with blood neutrophils, and most of these genes were down-regulated in sputum neutrophils, while the contrary was found for blood neutrophils in the exacerbation status. These data point to a different transcriptome profile for airway neutrophils as compared to that of circulating neutrophils, giving strength to the observation that the airway environment is causative of reprogramming of extravasated neutrophils in CF [17], [23]–[25], but are not consistent with results obtained with only 1050 genes by Adib-Conquy et al. [26]. Moreover, this difference was seen in both pre-therapy and post-therapy neutrophils (Figure 6), suggesting that antibiotic treatment does not cause a profound modification in gene expression of extravasated neutrophils. Nevertheless, the three genes object of this study had the same trend as in blood neutrophils (Figure 4), and this should be further investigated in light of correspondence between circulating and airway neutrophils. However, here we have found that airway and blood neutrophils expressed a common limited set of 206 genes (Table S5). A comparison with expression in control blood neutrophils revealed that airway and blood neutrophils showed also a difference in the variance of these 206 genes (Figure 6), again suggesting a transcriptome profile of airway neutrophils not coincident with that of blood neutrophils. Interestingly, antibiotic therapy did not substantially change this pattern (Figure 6), indicating a non responsiveness of airway neutrophils, likely due to lack of therapeutic amounts of drug in the lung.

There are a few important limitations to this work. First, this study was done on a small cohort. Validation of the results reported herein will need a higher number of patients, either homozygous for the F508del mutation or bearing other mutations. Moreover, some patients of our study did not respond to the intravenous antibiotic treatment. We have not excluded nonresponders, which would have further limited the consistency of the study. Second, we did not compare patients in acute exacerbation with patients in stable conditions, thus we could not identify the baseline gene expression profile. Nevertheless, these results should be further expanded to comprehend whether these genes have applicability as biomarkers in clinical trials for antibiotics and anti-inflammatory drugs.

## Conclusions

Genome-wide analysis of gene expression might be a novel tool for identifying novel genes and pathways which are modified in CF patients upon acute exacerbation conditions and sensitive to treatment. Blood neutrophils are easy to obtain allowing the inclusion of those patients which cannot expectorate. Three neutrophil genes in particular (*ARRB1*, *PMAIP1* and *HVCN1*) are possible candidates and their functional alteration should be considered in the context of pathways involved in granule exocytosis, apoptosis and respiratory burst.

## Supporting Information

Table S1 **Utilization of RNA samples obtained from blood and airway CF neutrophils.**
(DOCX)

Table S2 **Genes differentially expressed in blood neutrophils obtained from CF patients before therapy relative to control subjects at a fold change threshold of 1.4.**
(XLS)

Table S3 **Genes differentially expressed in blood neutrophils obtained from CF patients after therapy relative to the same patients before therapy at a fold change threshold of 1.4.**
(XLS)

Table S4 **Genes differentially expressed in sputum neutrophils obtained from CF patients after therapy relative to the same patients before therapy at a fold change threshold of 1.4.**
(XLS)

Table S5 **Genes differentially expressed between blood and airway neutrophils.**
(DOCX)
