# Supplementary material for: Evaluation of Genome-Wide Expression Profiles of Blood and Sputum Neutrophils in Cystic Fibrosis Patients Before and After Antibiotic Therapy
Source: PLoS One. 2014 Aug 1;9(8):e104080. doi: 10.1371/journal.pone.0104080 (PMC4118979; doi:10.1371/journal.pone.0104080)
Supplement: Table S1 — Utilization of RNA samples obtained from blood and airway CF neutrophils. (DOCX) [file pone.0104080.s001.docx]

Table S1. Utilization of RNA samples obtained from blood and airway CF neutrophils.

| **Patient #** | **Microarray on blood neutrophils** | **Real-time on blood neutrophils** | **Microarray on airway neutrophils** | **Real-time on airway neutrophils** |
| --- | --- | --- | --- | --- |
| 1 | √ |  |  |  |
| 2 | √ |  |  | √ |
| 3 | √ | √ | √ |  |
| 4 | √ |  |  |  |
| 5 | √ |  |  | √ |
| 6 | √ | √ | √ |  |
| 7 | √ | √ |  |  |
| 8 | √ | √ |  |  |
| 9 | √ (used only as pre-therapy sample) |  |  | √ |
| 10 | √ (used only as pre-therapy sample) |  | √ | √ |
| 11 |  | √ |  |  |
| 12 |  | √ |  | √ |
| 13 |  | √ |  |  |
| 14 |  | √ |  |  |
| 15 |  |  | √ | √ |
| 16 |  | √ |  |  |
