# Supplementary material for: Evaluation of Genome-Wide Expression Profiles of Blood and Sputum Neutrophils in Cystic Fibrosis Patients Before and After Antibiotic Therapy
Source: PLoS One. 2014 Aug 1;9(8):e104080. doi: 10.1371/journal.pone.0104080 (PMC4118979; doi:10.1371/journal.pone.0104080)
Supplement: Table S5 — Genes differentially expressed between blood and airway neutrophils. (DOCX) [file pone.0104080.s005.docx]

Table S5. Genes differentially expressed between blood and airway neutrophils.

| **Biological process** | **Gene ID** |
| --- | --- |
| Actin cytoskeleton  Amino acid transport  Apoptosis  Blood coagulation  Cell adhesion  Cell cycle  Cell differentiation  Chromosome segregation  Cilia formation  Complement  DNA repair  Exocytosis/endocytosis  Hormone receptor | AHNAK, ANKRD36, ANKRD36B, CD2AP, CFL2, LRPPRC, SDAD1, SRGAP2  SLC38A1  API5, DNM1L, FASTKD2, IFI6, PPP3CC, TNFSF10, XAF1  GP1BB, LMAN1, SERPINB2  CD36, ITGA4, ITGB1, THBS1  ANAPC1, BUB3, CCNC, DUSP10, FYN, MPHOSPH9, NAP1L1, PPP1CC, TOB1  NELL2, PLAC8, SLFN5, TTC3  HAUS6  IFT80  SERPING1  RFC1, SMC4  S100A10  NR1D2, RORA |
| Immune response  Interferon response | ABCE1, CCR7, CD3G, CD96, CD99, C19orf59, HLA-DQA1, HLA-DQB1, HLA-DRB5, HSP90AB1, IFIT1, IGJ, IL4R, IL7R, KLRC3, KLRC4, KLRK1, OSM, PTGER2, RSAD2  IFNG, MX1, OAS1, OAS3 |
| Inflammation  Ion channel  Metabolism  Metal transport  Mitochondrial electron transport  Nucleosome assembly  Protein modification/trafficking  Protein translation  Proteolysis  Purinergic receptor  RNA processing | ANXA1, CYSLTR1, HERC5, IL18R1, PPBP  ATP1B1  ALG9, ALPL, APOL6, ARG1, BCAT1, GCLC, GYG1, IMPAD1, INPP4B, LDHB, LPIN1, PYROXD1, ST3GAL4, SUCLG2  SLC39A10  NDUFA5  HIST1H1C, HIST1H1E, HIST1H4E, HIST2H2AA3, HIST2H2BF  CALU, DDHD2, DNAJC10, FRMD4B, IPO11, SSR3  DDX1, EIF3E, EIF3M, EIF5B, EPRS, PTCD3, RPL5, RPL6, RPL10A, RPL15  CAPN2, PREPL  P2RY13, P2RY14  KRR1, MBNL2, METT10D, MPHOSPH10, NGDN, RSL1D1, SKIV2L2, SNRPA1, SNRPN, TYW3, UTP15, WDR36, WDR43, WDR75, XPO1 |
| Signal transduction  Transcription factor  Transcription regulation  Ubiquitination  Unknown | ACVR2A, AKT3, ANXA2, ANXA3, ARHGEF3, ARL4C, GBP1, GBP3, GBP4, KIAA0748, MAP4K3, MAP4K5, MS4A7, PLEKHA1, PRKACB, RAP1GDS1, RASGEF1B, RASGRP1, RCAN3, SH2D1A, SMAD3, STAT4, TRAT1, TXK  AHR, EGR1, ETS1, FOS, LEF1, TFAM, ZBTB38, ZNF383, ZNF91  CAMK4, KLF12, MIER3, PTMA, RBAK, RPRD2, RRN3, TCERG1, TTC37, ZNF507  FBXO32, RNF125, USP10, WWP1  ATMIN, BMI1, C5orf33, CBLB, CCDC59, CCDC6, CCDC88C, CLIP4, CUL5, CYorf15B, DDX60, DENND4C, EFTUD1, EHHADH, EPSTI1, GCNT4, GLTSCR2, GPR183, GPR84, IFI44, IFI44L, IFIT3, MOP-1, PAR5, SNORD26, SNORD45A, TC2N, TTC39B, ZFAND1 |
